# Introducing new journal: Advances in Ophthalmology Practice and Research

**DOI:** 10.1016/j.aopr.2021.100002

**Published:** 2021-08-20

**Authors:** Ke Yao, Richard L. Abbott

**Affiliations:** Eye Center, Second Affiliated Hospital of Zhejiang University, School of Medicine, Eye Hospital of Zhejiang University, 88 Jiefang Road, Hangzhou, 310009, China; Wayne and Gladys Valley Center for Vision, University of California San Francisco, 490 Illinois Street, San Francisco, CA, 94158, USA

The WHO issued the first World Report on Vision in October 2019. According to this report, at least 2 billion people are living with vision impairment or blindness. Ophthalmic disease and vision impairment have created a significant global disease burden that continues to increase. Ophthalmologists worldwide face on-going challenges in the diagnosis and management of complex eye diseases. It is therefore critical that we share information and join global efforts to expand our clinical and applied research knowledge to protect visual health.

Due to the unique structure and intricate visual pathways of the eye, ophthalmic research is inevitably integrated with the principles and techniques of other related disciplines. These include materials science, tissue engineering, biomechanics, neuroscience, medical imaging, computer techniques, etc. With the rapid development of ophthalmology in recent years, many novel and innovative technologies, cutting-edge discoveries, and interdisciplinary research have emerged.

Ophthalmologists and basic scientists need an advanced, accessible, academic platform that integrates these multiple disciplines and encourages in-depth academic communication. A new journal, ***Advances in Ophthalmology Practice and Research (AOPR)****,* has been launched to help meet these needs.

AOPR is an international peer-reviewed open-access scientific journal, which is co-published by Elsevier and Zhejiang University Press on behalf of Zhejiang University and the Second Affiliated Hospital of Zhejiang University, School of Medicine. The Journal will publish clinical and basic research articles in various subspecialties of ophthalmology, vision science, related interdisciplinary and translational medicine fields, and advanced eye-related technologies and research findings. Its goal is to provide an academic communication platform for ophthalmic clinicians and scientists to promote the development of ophthalmology-related scientific research, as well as publish advancements in clinical diagnostics and therapeutics in ophthalmology,. Ultimately, it is hoped that this new information will contribute to the improvement of overall ocular health. The journal will publish a variety of article types, including original research, review articles, short communications, practice guidelines, opinions, editorials, comments, correspondence, and meeting reports.

AOPR is committed to providing a forum for innovative research in ophthalmology and other related disciplines, as well as providing a platform for the development of emerging technologies and theories. The editors and members of the editorial board enthusiastically welcome original research and clinical insights from our global colleagues and will share your outstanding work through our Journal with colleagues from around the world.

The successful launch of AOPR is dependent on the commitment and hard work of many people. We would like to acknowledge the hard work of all the editors, members of the editorial board and the staff of both Zhejiang University Press and Elsevier. We will continue making every effort to build AOPR into a world-class academic journal by providing a trusted, high quality international academic platform that promotes the advancement of ophthalmic science and related technology.

We look forward to receiving your contributions to AOPR, and welcome your comments and ideas on how to make this an outstanding journal. We can be reached through the Editorial Office (aopr@zju.edu.cn).

**Figure:**
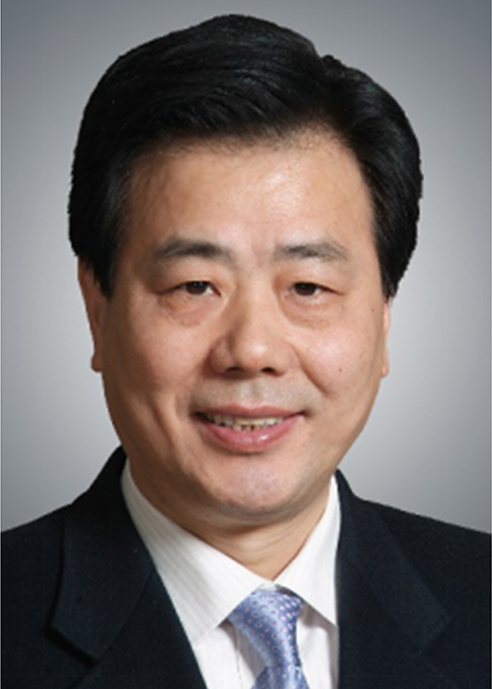
Ke Yao

**Figure:**
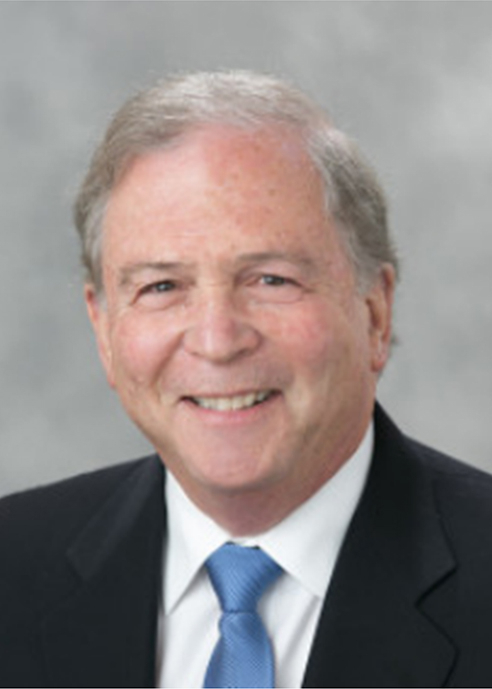
Richard L. Abbott

